# Help from the sky: Can vultures contribute to Cystic Echinococcosis control in endemic areas?

**DOI:** 10.1371/journal.pntd.0009615

**Published:** 2021-07-15

**Authors:** Fiammetta Berlinguer, Fahad Ahmed, Claudia Tamponi, Silvia Carta, Antonio Scala, Maria Grazia Cappai, Antonio Varcasia

**Affiliations:** Dipartimento di Medicina Veterinaria, Università degli Studi di Sassari, Sassari, Italy; University of Bari, ITALY

## Abstract

Cystic echinococcosis (CE) is endemic in Sardinia and constitutes a serious public health concern due to high prevalence in livestock and humans. Despite sustained efforts, control of the disease had been unsuccessful in the region. Problematic carcass disposal due to soaring incineration costs and free access of dogs to infected carrion are dominant factors, fueling endemicity among other. As sole obligate scavenger, griffon vultures (*Gyps fulvus*) are uniquely specialized to eliminate carcasses swiftly and efficiently, saving on unnecessary environmental and economic costs for carrion disposal. However, following drastic population declines across Europe, griffon vultures practically went extinct in Italy. A conservation expansion program in Sardinia successfully reinforced the last remaining Italian vulture population by mitigating the main threats to its survival; food shortage. Through the establishment of supplementary feeding stations, permanent supply of livestock cadavers was provided. In this research, the management and conservation implications on the controlled disposal of carcass disposal through vulture feeding stations on the control of CE in Sardinia were assessed. During the course of the project, vultures scavenged a total of 81,361 kg of biomass, saving €90,041 in incineration costs and € 1,054 in CO_2_ emission. Through extrapolation of these results, a total of 5,304 kg of suspected CE infected sheep carcasses (65.3%) was calculated to have been disposed by griffons, considerably reducing the CE risk and burden in Sardinia. A quantification of the amount of biomass that could be eliminated by griffon in a succeeding conservation project was also made. These calculations implied that 162,722 kg of biomass, including 10,608 kg of infected biomass from sheep, would be consumed over a period of 5 years, further lowering the CE burden in Sardinia. Our results, driven under one health approach, emphasize the crucial and direct role of griffons in breaking the lifecycle of CE as well as their indirect role in rendering multiple ecosystem and economic services through the elimination of carcasses. Please view a video Abstract here: https://youtu.be/Tm820nPq5KE.

## Author summary

Cystic echinococcosis (CE), is a major parasitic disease of animal and public health significance due to high morbidity and mortality. The infection is caused by the larval stage of the dog tapeworm *Echinococcus granulosus*, frequently reported in rural pastoral regions. In Sardinia, CE is very abundant in livestock, especially in sheep (65.3%). Several control strategies were attempted to limit the spread of CE, however, it turns out that the elimination of disease requires long lasting efforts. High prevalence of CE in Sardinia is due to multiple factors, but the cost of carcass incineration and free access of dogs to unattended carcasses are major points in the persistency of the infection. Griffon vultures as sole obligate scavengers, are highly specialized in removing the animal carcasses swiftly and efficiently, but have suffered severe population declines in the last decade. A wildlife conservation project in Sardinia envisioned to expand the griffon population by utilizing animal carcasses of animals as feed for vultures. We aimed to investigate these natural scavenging habits of vultures and the use of such “vulture restaurants” in relation to the control of CE. Our results show that the vultures remove a significant quantity of infected biomass, simultaneously saving high cost for incineration and carbon emission. The study is one of the pioneer investigations in the biological control of CE along with its estimation of ecosystem and economic services provided by the griffons.

## Introduction

Cystic echinococcosis (CE) is a major parasitic zoonotic disease of veterinary and public health significance distributed worldwide [1]. CE is considered as one of the neglected tropical helminth diseases for which effective surveillance measures are integral to curb the disease [1, 2]. CE is caused by infection with the members of the *Echinococcus granulosus sensu lato* (*s*.*l*.), species complex frequently reported in sheep-pastoral regions [3, 4]. The parasites have a two host predator-prey transmission cycle where canids serve as the definitive hosts as they harbor the adult worms in the small intestine. The tapeworm eggs are released in the environment via canids’ feces. Domestic ungulates, which accidentally ingest eggs from contaminated environment serve as intermediate hosts developing larval forms (cysts) in different organs, such as liver and lungs [5, 6]. Humans are generally considered as dead-end hosts and they are infected either by consumption of contaminated food or by direct contact with infected dogs [1, 2]. The disease is of special economic concern in terms of its implication on human health as well as livestock meat industry concomitantly inducing up to $ 3 billion monetary loss annually [7].

Cases of CE are commonly reported in various regions of the Mediterranean basin, including the island of Sardinia. As an established endemic focus of CE, Sardinia has the highest incidence of infection in the region involving different livestock species such as sheep, with an incidence ranging between 65.3%-75% [2, 8]; cattle, 9.4%-19.5% [9–11]; pigs, 9.4–11.1% and wild boars, 3.7% [3, 4].CE is regarded as a significant concern for public health in Italy, with an average annual incidence of 1.4 per 100,000 inhabitants [2]. At the species level, three species of *E*. *granulosus s*.*l*. complex targeting different hosts have been isolated in Sardinia, which include mainly the zoonotic species *E*. *granulosus sensu stricto (s*.*s*.*)*, and also *E*. *equinus*, and *E*. *canadensis* [3, 8]. Such stable endemism is associated with many socioeconomic and cultural factors however, unsupervised home slaughtering and improper disposal of carcass are the key factors in the continuous persistence of infection [2, 5]. On top of that, extensive farming practices where different animals species come in close contact, along with inadequate knowledge of the disease and the practice of feeding rural dogs with raw viscera from the slaughtered animals, are pivotal for spreading CE infection [5].

In the past, several epidemiological and surveillance programs were implemented to enforce direct preventive measures for CE, but they did not result in considerable technological improvements despite the fact that fundamental demographic, cultural and socioeconomic events happened in Sardinia [12]. The low profit margins on meat, together with the high costs of animal transport to abattoirs and incineration rates for carcasses are central factors underlying any major breakthrough in the fight against CE [12, 13]. Furthermore, the common but illegal practice of abandonment of carcasses in the remote areas allows free access of stray dogs to carrions contributing to persistence of CE in Sardinia.

In Sardinia, a strategic conservation project entitled "LIFE Under Griffon Wings" (LIFE14 NAT/IT/000484) funded by the European Union under the LIFE program was initiated in 2015 aiming to increase the status of the griffon vulture (*Gyps fulvus*) population in Sardinia. Under this project, a network of supplementary feeding stations was established in order to mitigate overall food shortage. One of the main limiting factors for vulture conservation in Europe is indeed the availability of safe food, due to changes in its geographic occurrence, quality and unavailability as a result of changes in European sanitary policies [14, 15] and the repeated poisoning events [16]. The griffon vulture population in Europe is significantly increasing, and it is estimated at 32,400–34,400 pairs [17], with Spain alone accounting for an estimated 30,000 pairs [18]. Its range has also expanded, thanks to reintroduction projects in France, the Italian peninsula and the Balkans [19]. However, in Italy the griffon vulture is still included on the Red List as ‘Near Threatened” [20], with the last natural population persisting on the island of Sardinia. In the beginning of the 20th century, Sardinia hosted a sizeable population of large raptors, with an abundance of griffon vultures, 150 pairs of black vultures (*Aegypius monachus)* and 35 pairs of bearded vultures (*Gypaetus barbatus)* [21]. However, their number quickly declined, and in the early 1960s, both the bearded and black vulture were declared extinct on the island. The population of griffon vultures was reduced to 25–30 reproductive pairs only [22]. Presently, the griffon vultures inhabit the north-west-coast of Sardinia between the municipalities of Bosa and Alghero [23].

Griffon vultures are highly specialized feeders capable of consuming large quantities of carrions. Given that, putrefying carcasses serve as a potential reservoir for various hazardous pathogens, these animals play a crucial ecosystem functions by removing carcasses from the environment. By doing so, vultures prevent the access of potential vectors e.g. feral dogs and wild boars to carrion, breaking the chain of infectious diseases [24–26]. Besides, their services allow to save on the cost of carcass removal [15, 27].

**Fig 1:**
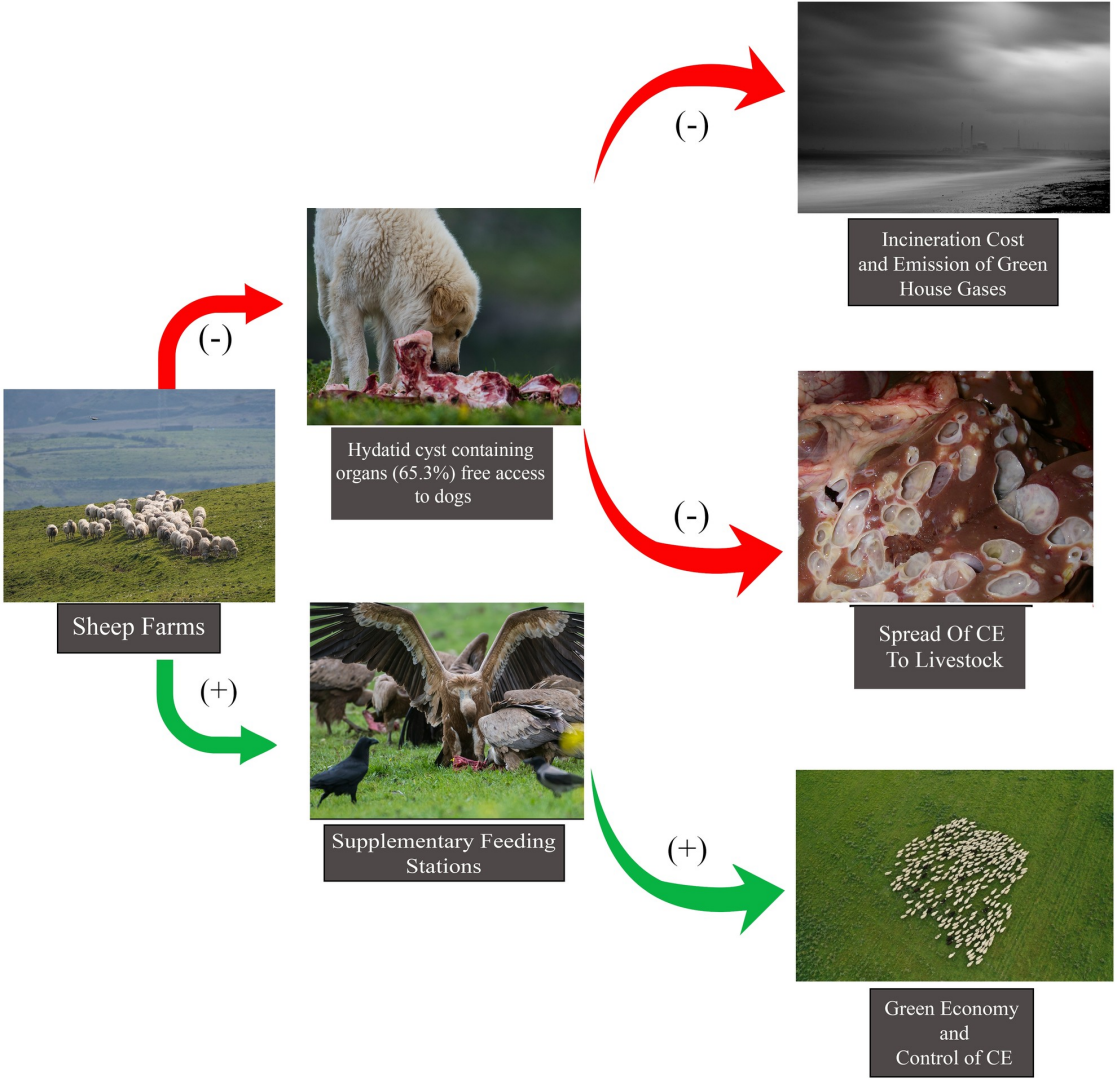
Graphical Abstract. Disruption of CE lifecycle by disposal of carcasses in vultures feeding stations and parallel ecosystem and economic services.

Considering the current expansion of the Sardinian vulture population and their efficient role in carcass elimination, this research envisions to determine if on site carrion disposal through vulture feeding stations could, alongside wildlife conservation efforts, indirectly benefit the efforts to combat CE (Fig 1). Specifically, could the decrease in the hazardous biomass (i.e. sheep offal) in extensive farming contribute to break the lifecycle of *E*. *granulosus s*.*l*. in Sardinia.

## Methods

### Ethics statement

No ethical approval was requested as the present study did not involve any invasive procedure, animal handling or manipulation.

### Study area and species

The experimental activities were carried out within the conservation project for griffon vultures, from 2015 to 2020 on the island of Sardinia, Italy. The island constitutes a major biodiversity hotspot in the Mediterranean basin including a vast number of indigenous species [28–30], some of which are classified as “endangered taxa”[31]. The landscape of Sardinia shows geomorphologic variability with hilly areas covering large portions of the island followed by flat land and a small series of mountainous areas [32]. The climate is characterized by mild to hot and dry summers with wet and cold winters and has an average annual rainfall of 756 mm [33]. The rural landscape is dominated by millennia of extensive livestock activities [34, 35] and the island currently holds approximately 2,896,905 head of sheep, representing 4% of the total European ovine population [36]. Such vast livestock numbers lead to large amounts of carcasses, capable of sustaining a sizeable avian scavenger population. The griffon vulture is a large, long lived, colonial breeder found in open hilly areas with high altitude cliffs typically abundant in the Mediterranean region particularly in the Western Palearctic [37].

### Establishment of Supplementary Feeding Stations (SFS)

Supplementary feeding stations or “vulture restaurants” provide a reliable food source for raptors worldwide and are a well-established tool employed for the conservation of griffon vultures [38, 39]. However, when feeding supplementation is managed as a limited number of heavy feeding stations, it poses several negative ecological effects [40], such as density-dependent reduction of productivity [41]; buffering effects on dispersal strategies, making birds stay in the natal population as a consequence of conspecific attraction; or even effects on trophic cascades upon small herbivores through facultative scavengers [42]. Therefore, light and dispersed feeding stations should be prioritised [43–46]. The feeding stations employed within the “LIFE Under Griffon Wings” project in Sardinia were installed in Nature 2000 network protected areas: Bosa, Capo Marargiu (Special Protection Area for Birds ITB023037) and Porto Tangone (Site of Community Importance SCI ITB020041) (Table 1). These municipalities were specifically chosen owing to the presence of geographical hallmarks and features propping vulture population e.g. elevated cliffs and rocky blocks used for nesting, breeding and roosting. Additionally, two centralized feeding stations within vulture foraging areas, in Porto Conte and Monte Minerva, were installed providing a reserve food depot. The feeding stations established in Sardinia were of distinctive nature as they were authorized and created within the farms by the Official Veterinary Service and directly managed by the farmers themselves under the supervision of an official veterinarian (https://www.atssardegna.it/) and LIFE project’s team. The feeding stations were surrounded by an electric fence, preventing the access of canids to carrion and offal. Alongside this, camera traps were installed on each feeding station for consistent monitoring of the feeding activities and also farmers were ensured to comply with all the regulations regarding disposal by the sanitary authorities. All feeding stations were associated with sheep, goat and bovine farms and to a lesser extent, equids. The carrion provided during the course of this survey consisted of dead carcasses from ovine/caprine (n = 255), bovine (n = 171) and equines (n = 2) origin Table 2 as a prey species distributed homogenously between all supplementary feeding stations across the region. The origin of all carcasses deployed can be found in Table 2. As feeding stations were set up inside farms, transportation of small animal carcasses was accomplished by a wheelbarrow, while large animal carcasses were transported via tractors to the feeding stations. The sanitary condition of the carcasses was verified with the help of breeders and landowners within the project areas. All personnel were first trained by the official veterinarians on the role of vultures and correct management of feeding stations, solely depositing animal carcasses that are free of drugs. Beside this, training on the prevention of the diffusion of diseases and zoonosis from infected carcass and the positive role of vultures in ecosystem was also stipulated. The presence of any drug residues in carcasses was also periodically inspected by the Istituto Zooprofilattico Sperimentale della Sardegna (IMHO).

**Table 1:** Carrion biomass in kg delivered from farms in each municipality and total number griffons fed with in the conservation project.

Feeding stations	Municipality	Total biomass provided in kg during the course of project	Total griffon fed from each municipality
1	Bosa	890	4.88
2	Bosa/Montresta	21,653	118.65
3	Bosa	13,660	74.85
4	Montresta	610	3.34
5	Montresta	21,420	117.37
6	Macomer	5,044	27.64
7	Macomer	3,220	17.64
8	Macomer	360	1.97
09	Bortigali	350	1.92
10	Bosa	295	1.62
11	Macomer	1,589	8.71
12	Macomer	40	0.22
13	Sindia	620	3.40
14	Sindia	6,610	36.22
15	Villanova Monteleone	1,540	8.44
16	Villanova Monteleone	425	2.33
17	Pozzomaggiore	1,350	7.40
18	Pozzomaggiore	125	0.68
19	Villanova Monteleone	885	4.85
20	Pozzomaggiore	500	2.74
21	Villanova Monteleone	85	0.47
22	Pozzomaggiore	90	0.49
Total	Municipality	81,361kg	445 griffons

**Table 2:** Total number of animal cadavers from each animal species and corresponding biomass produced with the cost saved from incineration and carbon emission in each year.

Year	Total animals	Biomass from bovines (N = 171)	Incineration cost saved bovines (0.97€/kg) [54]	Biomass from sheep/goat (N = 255)	Incineration cost saved sheep/goat (2.12€/kg) [54]	Biomass from equines (N = 2)	Incineration cost saved equines (1.07€/kg) [54]	Total Incineration cost saved/year	Total biomass consumed by griffons/year	CO2 cost saved/year (1tonne = 13€) [56]
2017	89	(N = 59) 21,993kg	21,333€	(N = 30) 1,206kg	2,556€	-	-	23,889€	23,199kg	300€
2018	110	(N = 38) 16,610kg	16,111€	(N = 71) 2,655kg	5,628€	(N = 1) 150kg	160.5€	21,899€	19,415kg	252€
2019	174	(N = 53) 23,010kg	22,319€	(N = 120) 4,598kg	9,747€	(N = 1) 500kg	535€	32,601€	28,108kg	365€
2020	55	(N = 21) 9,480kg	9,195€	(N = 34) 1,159kg	2,457€	-	-	11,652€	10,639kg	137€
Total	428	71,093kg	68,958€	9,618kg	20,388€	650kg	695.5€	90,041€	81,361kg	1054€

### Available biomass for griffons

The Eurasian griffon vulture (average body weight, BW: 9 kg) on average requires 500 g of carcass tissues per day [47], which represents a capacity of daily dry matter intake capacity of 1.6% of total bodyweight. The total biomass supplied through all feeding stations and biomass consumed by the griffon vultures was also calculated (Table 1). To estimate the biomass availability for the vultures, the average carcass weight of each species of livestock cadaver was estimated [48] and the following conversion formula was as used: livestock species*Mortality rate/100.

The carcass of livestock species was multiplied by a standard body weight: 500 kg for cattle [49] and 30 kg for goats and sheep [50] and a correction factor of 0.66 was applied. The total griffon vultures fed per year were calculated by dividing the biomass per year multiplied with daily feed intake of griffon vultures. We also ascertained the total amount of carcasses delivered to feeding stations per year and the total amount of biomass annually consumed by vultures (Table 1).

As griffon vultures forage meat as well as animal byproducts, the biomass available to vultures is 31% of the total body weight of cattle [51, 52] and 27% of goat and sheep [53]. The amount of biomass produced from individual animal species was also calculated during the time frame of the project (Table 2). In Sardinia, the food resources of the species consist mostly of carcasses of reared animals provided by extensive and semi-extensive farms; the sheep and goats represent over 80% of its diet in particular [22, 23]. Data on carrying capacity have been calculated considering data on number and consistency of farms provided by the Regional Veterinary Epidemiological Observatory compared with the expected increase in the griffon vulture population. The mortality rate was estimated to be 8% for the small animals and 5% for large animals [52, 53].

### Ecosystem services

To estimate the economic balance value of the animal waste removal service provided by the griffon vultures, the costs of incineration and animal waste disposal from the processing plant of the Ministero delle politiche agricole were used [54]. Following, vulture feeding stations would save 0.97 €/kg, 2.21 €/kg and 1.07 €/kg on the disposal (including the cost for transportation) of bovine, sheep/goat and equine carcasses respectively [54]. As carcass incineration as well as the transport of said carcasses to waste disposal plants generate large amount of greenhouse gases (GHG) (e.g. nitrous oxide, methane and carbon dioxide) impacting the environment and accelerating global warming [55], the cost saved based on reduced CO_2_ emission through the feeding of carrion to vultures was evaluated. A recent reference study estimated the cost of 1 ton of CO_2_ produced through incineration to amount to € 13 [56]. Expenditure saved by vultures was calculated by simply converting the total biomass consumed into tonnes.

## Results

A total of 199 carcasses were available in the GCL study area, with a combined biomass of 22,957 kg.

A total of 199 carcasses were available in the GCL study area, with a combined biomass of 22,957 kg.

A total of 199 carcasses were available in the GCL study area, with a combined biomass of 22,957 kg.

In total, 81,361 kg of biomass originating from cattle, sheep, goat and equine carcasses was consumed by vultures during the course of the conservation project (Table 1); 23,199 kg in 2017, 19,415 kg in 2018, 28,108 kg in 2019 and 10,639 kg in 2020 (Tables 1 and 2). Specifically, 8,123 kg of sheep, 1,495 kg of goat, 71,093 kg of cattle and 650 kg of equine carrion was consumed corresponding to 255, 171 and 2 individuals respectively (Table 2). In view of griffon vulture daily food intake, which is 0.5 kg per animal [47], the total number of vultures fed/year from each municipality was calculated, which summed up to 445 individuals during the course of the project (Table 1). Being a scavenger, the vulture’s diet includes animal carcasses, which may potentially be infected with various infectious pathogens [57]. However, the vultures have evolved a robust capacity to cope with these pathogenic organisms. They possess a highly specialized digestive system, strong immune system and stable intestinal microbiome to counter these pathogens [58, 59]. The prevalence of CE is estimated to be 65.3% in sheep in Sardinia [2] which indicates that about 5,304 kg of infected carrion is consumed by vultures thereby reducing the burden of CE in Sardinia drastically. Even though, five (5) carcasses were tested positive for drugs, no noteworthy quantities of veterinary pharmaceuticals were encountered.

The annual monetary value of the animal waste removal service provided by vultures was € 90,041, corresponding to savings of € 68,958 on bovine, € 20,388 on sheep and goat and € 695.5 on equine carcass transport and incineration costs (Table 2). These values also include transportation cost, and this is particularly important in an island like Sardinia.

The total estimated cost saved based on atmospheric CO_2_ emission was € 1,054; € 300 in 2017, € 252 in 2018, € 365 in 2019 and € 137 in 2020 (Table 2). This showed that the ecosystem services provided by the vultures extend not only to saving incineration costs, but also unnecessary environmental pollution.

The succeeding project “LIFE Safe for Vultures” was initiated in 2021, to expand the feeding station network to the whole island of Sardinia, while until now feeding stations were built only in the places where actually most of the griffons reside. The aim of the new LIFE project is to favor the return of vultures to all historical regions of Sardinia and this process will be boosted also by the translocation of another 50 vultures in the south of the island. We anticipate total vulture consumption to double (162,722 kg of organic matter) during the “LIFE Safe for Vultures” project. Moreover, the consumption of 16,246 kg of sheep carrion is expected, including an estimated 10,608 kg of infected biomass. Costs saved on transport and incineration are estimated at € 180,082 and € 2,115 based on CO_2_ emission reduction.

If all of these carcasses were made available to the griffon vultures a total biomass of 87,825 tonnes/year would be provided, meeting the the requirements of 4,812 griffons/year. Hence, livestock carrion biomass would not be a limiting factor for the population growth if all livestock carcasses within the core foraging range were made available to the vultures.

## Discussion

Our results strongly underscore the role of vultures in carrion disposal, and the service they provide by both controlling and limiting the spread of CE through the disruption of parasite’s lifecycle, preventing the access of scavengers to infected carcasses. Based on our findings, we established that griffon vultures remove a substantial quantity of carrions yearly, which is often challenging due to high transport and incineration costs. Additionally, vultures contribute substantially to greenhouse gas emissions reduction.

Sardinia is considered an endemic hotspot for CE with a disease prevalence of 65.3% in sheep [2]. High intensity of CE on the island most likely results from the unofficial slaughtering of farm animals and abandonment of carcasses in remote areas [5]. Moreover, unsupervised home slaughter, feeding raw offal to dogs, and in particular the scanty record keeping leading to common disappearance of animals from livestock holdings and from production lines are daunting challenges, leading to hyperendemicity of CE in the region [2, 5, 60]. The dog population in Sardinia is estimated at 200,000 individuals, with a substantial farm dog (80,000) and stray dog (70,000) population, with 16–20% infection rate, demonstrating high spreading potential of the disease [12]. In Sardinia, almost all dogs have free access to carcasses for scavenging due to non-disposal and of deliberate feeding by farmers, which is a traditional malpractice. This can be verified by the fact that the mean culling rate of sheep reared in Sardinia is 15–20% per flock. Following at least 300,000–400,000 sheep are expected to be slaughtered each year, however, official figures of 150,000 do not correlate with the official data provided from 2002–2008 by ISTAT, which indicates that approximately half of the animals are scavenged by dogs [12]. A large number of animals are probably slaughtered at farms and many animals are declared missing, most likely dead on pastures [2]. A very important recommendation is put forward to prevent clandestine slaughtering is providing electronic identification to farm animals, because it is a requirement for farmers to have access to EU community funds. On the other hand, no incinerator is available/working for animal byproducts in Sardinia and therefore, all the carrions are shipped overseas for incineration, with related surplus costs due to ferry transportation.

Vultures are specialized obligate scavengers and are specialized in eliminating carcasses carrying very serious infectious diseases such as bovine tuberculosis and anthrax [61]. By doing so, these birds contribute, besides the redistribution of nutrients, to significant sanitary ecosystem services [27, 62, 63]. Similarly, parasites with zoonotic potential such as cestodes (e.g., *E*. *granulosus*) and protozoans (e.g., *T*. *gondii*) present in the carcasses can efficiently be scavenged by vultures, without any risk of transmission to humans and animals. It is an evident fact that CE cannot be eliminated by griffons, however, their potential scavenging behavior can facilitate disease regulation when till date all human attempts were futile and ineffective. Moreover, griffon can’t and will never be able to compete with dogs, because the feeding practices totally rely on farmers behavior. If by any chance there would be a competition between griffons and dogs, the dogs will outcompete every single time. However, the basic aim of our study was to highlight the scavenging by the right species, thus replacing dogs with griffons, which naturally keep the environment clean without any zoonotic risk. Although, the role of vultures as the reservoirs for multiple zoonotic pathogens is documented in various studies e.g. *Salmonella* and *Campylobacter* [40, 64–66]. However, no clear information exists on the epidemiological role of vultures in spread of the diseases to humans and animals [67]. Empirical data confirms that vultures decline would promote infectious diseases’ transmission to animals as well as humans in future [24, 27, 68–70].

The sympatric interaction between vultures and humans has existed since ancient times [71] and consists of a commensal and mutualist relationship providing valuable ecosystem services [72]. It is important to recognize that the vulture’s ecosystem services are not only limited to decreasing carrion burden, but also extend to economic benefits coupled with inhibiting emission of CO_2_ in the environment [37, 55, 73].Within this research, vulture feeding prevented the incineration of 81,361 kg of biomass which would otherwise have led to a substantial CO_2_ emission, causing environmental pollution with considerable ecological consequences. Economically, griffon vultures in Sardinia saved € 90,041 of public funds that would have been used for carcass disposal, which is substantially lower than the amount calculated for vultures in France [56]. Besides, as ecological tourism is currently thriving throughout the world, and given the changing public opinion on vultures in general, wildlife conservation activities in Sardinia could lead to significant economic gains in the future. Overall, a heightened interest in vultures can be noted in the context of bird watching and wildlife photography [24, 74, 75]. In Israel, the potential annual return from tourist visits specifically targeting griffon vultures was estimated at $ 1–2 million [76]. However, it should also be kept in mind that an increasing tourism will, in turn, also increase the carbon footprint massively again.

Extensive farming is a common practice in Sardinia and other Mediterranean countries and our results demonstrate that large amount of biomass produced by this system could aid in vulture conservation, but could also alleviate various other issues related to economics of farmers and disease control. The availability of carrion is unstable and pulsatile around the year for vultures [73, 77, 78] and this is especially important during the critical stages in which substitute food resources are unavailable. Alleviating food unavailability due to seasonal variations, as well as predatory challenges by creating supplementary feeding stations, is the measure advocated to rescue vultures in current study [37, 44, 79].

It is noteworthy that Sardinian transhumant communities have presently shifted to a moderately stable sheep farming system, leading to the increased tendency of farmers to invest in and exercise contemporary breeding operations. However, technological development in meat processing in order to comply with European regulations has considerably decreased the number of slaughter houses nowadays. Additionally, carcass transport and processing expenditures have increased enormously as only one incinerator remains for the whole of Sardinia, which, on top of that, is currently nonfunctional. Low profit margins on meat and compulsory inspection of animals by official veterinarians at abattoirs have drastically limited farmer’s investment capacity and promoted the practice of home slaughtering. In an effort to counter this, several measures were taken by the Regional Government of Sardinia including electronic registering of sheep in order to guarantee traceability and the induction of mobile abattoirs to dispose of offal and incentives to cover carcass transportation costs. Despite all, current disease trends imply all attempts in lowering CE infection in Sardinia have been futile [2, 12].

There are numerous advantages to the implementation of supplementary feeding stations for avian scavengers. However, various challenges are present as well such as the presence of veterinary drugs e.g. Diclofenac sodium, or other harmful chemicals e.g. (lead) in the carcasses [46]. From a practical point of view, spatial distribution of feeding stations cannot be generalized to a whole region and it requires a balance according to the griffon vultures’ population size.

In conclusion, due to the scavenging behavior and efficient ecosystem services provided by vultures, these birds could be of special significance in the fight against CE in Sardinia where incidence rates are very high. The correlation between vultures and disease prevention as highlighted in this research puts their conservation efforts within the “One Health” domain and, furthermore, offers a viable control method for the disease without any risk of spillover at the human-wildlife-livestock interface. The inferences from this study can be broadly generalizad to other islands such as Corsica. Finally, we believe that there is a dire need for the general public to acknowledge the essential but, neglected, ecosystem services provided by griffon vultures, which would diminish their negative image and thus promote their conservation.

## Supporting information

S1 Video Video Abstract, also available at https://youtu.be/Tm820nPq5KE.
(MOV)
